# *Pseudomonas aeruginosa* Susceptibility Patterns and Associated Clinical Outcomes in People with Cystic Fibrosis following Approval of Aztreonam Lysine for Inhalation

**DOI:** 10.1128/AAC.02327-20

**Published:** 2021-02-17

**Authors:** Claire L. Keating, Jonathan B. Zuckerman, Pradeep K. Singh, Matthew McKevitt, Oksana Gurtovaya, Mark Bresnik, Bruce C. Marshall, Lisa Saiman

**Affiliations:** a Division of Pulmonary, Allergy and Critical Care, Columbia University Irving Medical Center, New York, New York, USA; b Division of Pulmonary and Critical Care, Maine Medical Center, Portland, Maine, USA; c Department of Microbiology and Medicine, University of Washington, Seattle, Washington, USA; d Gilead Sciences, Inc., Seattle, Washington, USA; e Gilead Sciences, Inc., Foster City, California, USA; f Cystic Fibrosis Foundation, Bethesda, Maryland, USA; g Department of Pediatrics, Columbia University Irving Medical Center, New York, New York, USA

**Keywords:** cystic fibrosis, antimicrobial drug resistance, aztreonam, *Pseudomonas aeruginosa*

## Abstract

The approval of aztreonam lysine for inhalation solution (AZLI) raised concerns that additional antibiotic exposure would potentially affect the susceptibility profiles of *Pseudomonas aeruginosa* isolates from cystic fibrosis (CF) patients. This 5-year, prospective, observational study tracked susceptibility changes and clinical outcomes in CF patients in the United States with chronic *P. aeruginosa* infection. Sputum cultures were collected annually (2011 to 2016). The primary study endpoint was the proportion of subjects whose least susceptible *P. aeruginosa* isolate had an aztreonam MIC that was >8 μg/ml (parenteral breakpoint) and increased ≥4-fold compared with the least susceptible isolate from the previous year. Annualized data for pulmonary exacerbations, hospitalizations, and percent of predicted forced expiratory volume in 1 s (FEV_1_% predicted) were obtained from the CF Foundation Patient Registry and compared between subjects meeting and those not meeting the primary endpoint. A total of 510 subjects were enrolled; 334 (65%) completed the study. A consistent proportion of evaluable subjects (13 to 22%) met the primary endpoint each year, and AZLI use during the previous 12 months was not associated with meeting the primary endpoint. While the annual declines in lung function were comparable for subjects meeting and those not meeting the primary endpoint, more pulmonary exacerbations and hospitalizations were experienced by those who met it. The aztreonam susceptibility of *P. aeruginosa* remained consistent during the 5-year study. The relationship between *P. aeruginosa* isolate susceptibilities and clinical outcomes is complex; reduced susceptibility was not associated with an accelerated decline in lung function but was associated with more exacerbations and hospitalizations, likely reflecting increased overall antibiotic exposure. (This study has been registered at ClinicalTrials.gov under identifier NCT01375036.)

## Abstract (precis)

The approval of aztreonam lysine for inhalation solution (AZLI) raised concerns that additional antibiotic exposure would potentially affect the susceptibility profiles of *Pseudomonas aeruginosa* isolates from cystic fibrosis (CF) patients. This 5-year, prospective, observational study tracked susceptibility changes and clinical outcomes in CF patients in the United States with chronic *P. aeruginosa* infection.

## INTRODUCTION

Pseudomonas aeruginosa is the most common cause of lower respiratory tract infection in cystic fibrosis (CF) patients, affecting ∼50% by the age of 18 years (1). Chronic suppression of P. aeruginosa infection with inhaled antimicrobial therapies is a cornerstone of CF care (2) and is associated with reductions in the rates of exacerbations, hospitalizations, and decline in lung function (3, 4). However, chronic use of antipseudomonal antibiotics in CF patients poses some dilemmas. While antimicrobial resistance, due to continuous antibiotic exposure, is a concern (5), there is also ongoing debate regarding the clinical relevance of MIC values and parenteral susceptibility breakpoints for the management of chronic *P. aeruginosa* infections (6–10). Nonetheless, changing resistance patterns and potential associations of resistance with key clinical outcomes in CF patients remain important issues to investigate.

Aztreonam lysine for inhalation solution (AZLI) (Cayston; Gilead Sciences) (U.S. approval in 2010) is an inhaled monobactam antibiotic indicated to improve respiratory symptoms in CF patients with *P. aeruginosa* airway infection (11). As a postmarketing commitment to the U.S. Food and Drug Administration (FDA), this 5-year prospective study sought to address concerns that the introduction of AZLI may exert additional selective pressures, thereby decreasing the susceptibility of *P. aeruginosa* isolates. We also examined whether changes in susceptibility are associated with changes in clinical outcome measures using data obtained from the U.S. Cystic Fibrosis Foundation Patient Registry (CFFPR).

(This work has been previously reported in abstract form [12, 32].)

## RESULTS

In all, 510 subjects were enrolled, and 334 (65.5%) completed the 5-year study (see Fig. S1 in the supplemental material). At enrollment, the subjects’ mean age was 26 years (range, 6 to 71 years), and the mean annualized percent of predicted forced expiratory volume in 1 s (FEV_1_% predicted) for 2011 was 63.7% (Table 1). Characteristics were comparable for enrolled subjects and CFFPR patients with chronic *P. aeruginosa* infection (based on Leeds criteria) (Table 1) (13, 14). Most subjects (85%) reported the use of inhaled antibiotics in 2011, and 75% of *P. aeruginosa* strains isolated at baseline had the mucoid phenotype, consistent with the presence of chronic *P. aeruginosa* infection. During the 12 months before enrollment, 54% of subjects received AZLI (mean number of courses, 2.3). Lung function at enrollment (2011) differed for subjects who had used versus those who had not used AZLI (FEV_1_% predicted, 60.3% versus 67.7%). Fewer subjects receiving prior AZLI had mild lung disease (FEV_1_ ≥70% predicted, 29% versus 46% of subjects).

**TABLE 1:** Subject characteristics at enrollment compared to CFF Patient Registry data

Parameter	Value for group			
	Subjects with AZLI use in 12 mo prior to 2011 study enrollment		All study subjects (*n* = 510)	2011 CFF Registry patients with chronic *P. aeruginosa* infection^*a*^ (*n* = 8,084)
	Yes (*n* = 274)	No (*n* = 236)		
Mean age at 2011 enrollment (yrs) (SD)	26.0 (11.1)	25.9 (13.1)	26.0 (12.0)	27.2 (11.3)^*b*^
No. of subjects aged <18 yrs at 2011 enrollment (%)	68 (24.8)	65 (27.5)	133 (26.10)	1,681 (20.8)^*b*^
No. of subjects of gender (%)				
Female	153 (55.8)	121 (51.3)	274 (53.7)	3,901 (48.3)
Male	121 (44.2)	115 (48.7)	236 (46.3)	4,183 (51.7)
No. of subjects of race/ethnicity (%)				
White	266 (97.1)	225 (95.3)	491 (96.3)	7,662 (94.8)^*c*^
Black or African heritage	2 (0.7)	7 (3.0)	9 (1.8)	310 (3.8)^*c*^
Hispanic, other, or missing	6 (2.2)	4 (1.7)	10 (2.0)	501 (6.2)^*c*^
No. of patients with genotype (%)				
F508del homozygote	160 (58.4)	111 (47.0)	271 (53.1)	4,151 (51.3)
F508del heterozygote	92 (33.6)	83 (35.2)	175 (34.3)	3,019 (37.3)
Unidentified, other, or missing	22 (8.0)	42 (17.8)	64 (12.5)^*d*^	914 (11.3)
No. of patients exposed to inhaled antibiotics in 2011 (%)^*e*^			425 (84.5)	
Exposed to inhaled aztreonam in 2011^*f*^			264 (52.5)	
Exposed to inhaled tobramycin in 2011^*f*^			345 (68.6)	
Exposed to inhaled colistin in 2011^*f*^			65 (12.9)	
Mean annualized FEV_1_% predicted for 2011^*g*^ (SD)	60.3 (18.3)	67.7 (18.4)	63.7 (18.7)	64.0 (24.0)
No. of patients with lung disease severity^*g*^ (%)				(*n* = 7,962)
Mild (≥70% FEV_1_ predicted)	78 (28.5)	109 (46.2)	187 (36.7)	3,410 (42.8)
Moderate (40% to 69% FEV_1_ predicted)	142. (51.8)	92 (39.0)	234 (45.9)	2,996 (37.6)
Severe (<40% FEV_1_ predicted)	54 (19.7)	35 (14.8)	89 (17.5)	1,556 (19.5)

a Cystic Fibrosis Foundation (CFF) Registry data were described previously (13). Registry patients with chronic Pseudomonas aeruginosa infection by the Leeds criteria (14) are included in this column.

b Age at the end of 2011.

c Patients could select more than 1 category.

d Included 19 subjects (3.7%) with unidentified genotypes, 34 (6.7%) with other genotypes, and 11 (2.2%) with missing genotypes.

e Included inhaled tobramycin, colistin, or aztreonam, alone or in combination. Data were from the CFF Registry and were available for 503 subjects.

f Data were from the CFF Registry and were available for 503 subjects. Some subjects received >1 inhaled antibiotic. Note that the proportion of subjects recorded in the CFF Registry as receiving inhaled aztreonam in 2011 (52.5%) differs slightly from the proportion of subjects recorded in a study questionnaire as having used AZLI in the 12 months prior to study enrollment (53.7%).

g Annualized percent of predicted forced expiratory volume in 1 s (FEV_1_% predicted) data from the CFF Patient Registry.

Respiratory specimen collection and culture results are shown in Tables 2 and 3, respectively. *P. aeruginosa* was isolated and MIC data were available for 72 to 79% of annual samples. During the 5-year study, most specimens were sputum samples (72 to 84%). The median numbers (ranges) of morphologically distinct *P. aeruginosa* colonies per specimen were 1 (1 to 4) at baseline (2011) and 2 (1 to 5) for all subsequent study years, with 75 to 81% having the mucoid phenotype across the 5 years.

**TABLE 2:**
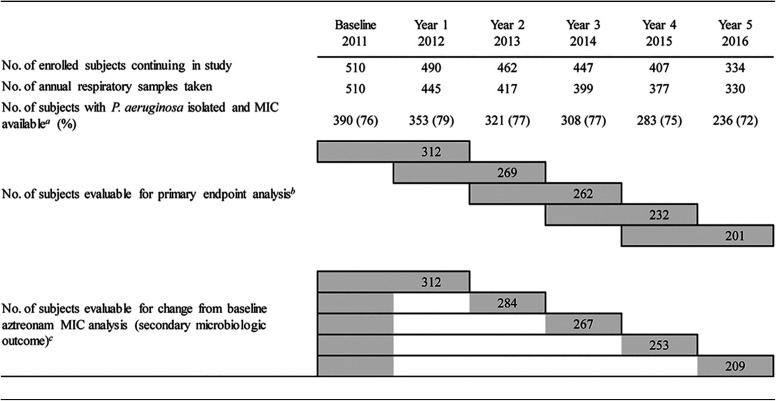
Subjects available for evaluation of microbiologic endpoints

a The denominator for the calculation of the percentage is the number of subjects with an annual sample.

b Evaluable subjects had aztreonam MIC data available from 2 visits, as indicated by the left- and righthand ends of the gray bars. For example, 262 subjects were evaluable at year 3 for the primary endpoint analysis because they had data available for year 2 and year 3. Note that subjects could move between AZLI use categories (yes/no for each year) across the study.

c Evaluable subjects had baseline data available and aztreonam MIC data from the relevant year. For example, 267 subjects were evaluable to assess the change from the baseline MIC to the year 3 MIC.

**TABLE 3:** Aztreonam MIC_50_ and MIC_90_ values for all Pseudomonas aeruginosa isolates

Parameter	Value at study visit					
	Baseline (2011)	Yr 1 (2012)	Yr 2 (2013)	Yr 3 (2014)	Yr 4 (2015)	Yr 5 (2016)
Subjects with *P. aeruginosa* present and MIC available at study visit in given yr^*a*^						
No. of subjects	390	353	321	308	283	236
No. of *P. aeruginosa* isolates	611	593	575	562	501	406
Aztreonam MIC_50_ (μg/ml)	4	4	4	4	4	8
Aztreonam MIC_90_ (μg/ml)	128	128	128	128	256	256

Evaluable subjects with *P. aeruginosa* present, MIC available, and AZLI use during 12 mo prior to the individual study visit^*a*^^,^^*b*^						
No. of subjects	229	199	193	178	159	126
No. of *P. aeruginosa* isolates	345	333	328	315	269	209
Aztreonam MIC_50_ (μg/ml)	8	8	8	8	8	16
Aztreonam MIC_90_ (μg/ml)	256	256	256	512	512	512

Evaluable subjects with *P. aeruginosa* present, MIC available, and no AZLI use during 12 mo prior to the individual study visit^*a*^^,^^*b*^						
No. of subjects	161	151	127	129	123	109
No. of *P. aeruginosa* isolates	266	256	245	246	231	195
Aztreonam MIC_50_ (μg/ml)	≤1	2	4	2	2	4
Aztreonam MIC_90_ (μg/ml)	32	32	64	64	64	64

a Aztreonam concentrations from 1 to 2,048 μg/ml were evaluated to determine MIC values. Overall, aztreonam MIC values ranged from ≤1 to >2,048 μg/ml for each year during the study. For subjects with AZLI use during a given year, the highest aztreonam MIC values were >2,048 μg/ml at baseline and for each study year. For subjects without AZLI use during a given year, the highest aztreonam MIC values were 2,048 μg/ml at baseline and 512, >2,048, 1,024, 1,024, and 2,048 μg/ml at years 1 to 5, respectively. Note that subjects could move between AZLI use categories (yes/no for each year) across the study.

b Data regarding AZLI use were not available for all subjects; therefore, the numbers of evaluable subjects with and without AZLI use are sometimes slightly smaller than the total number of subjects with *P. aeruginosa* present and MICs available in a given year.

### Primary microbiologic endpoint.

**FIG 1:**
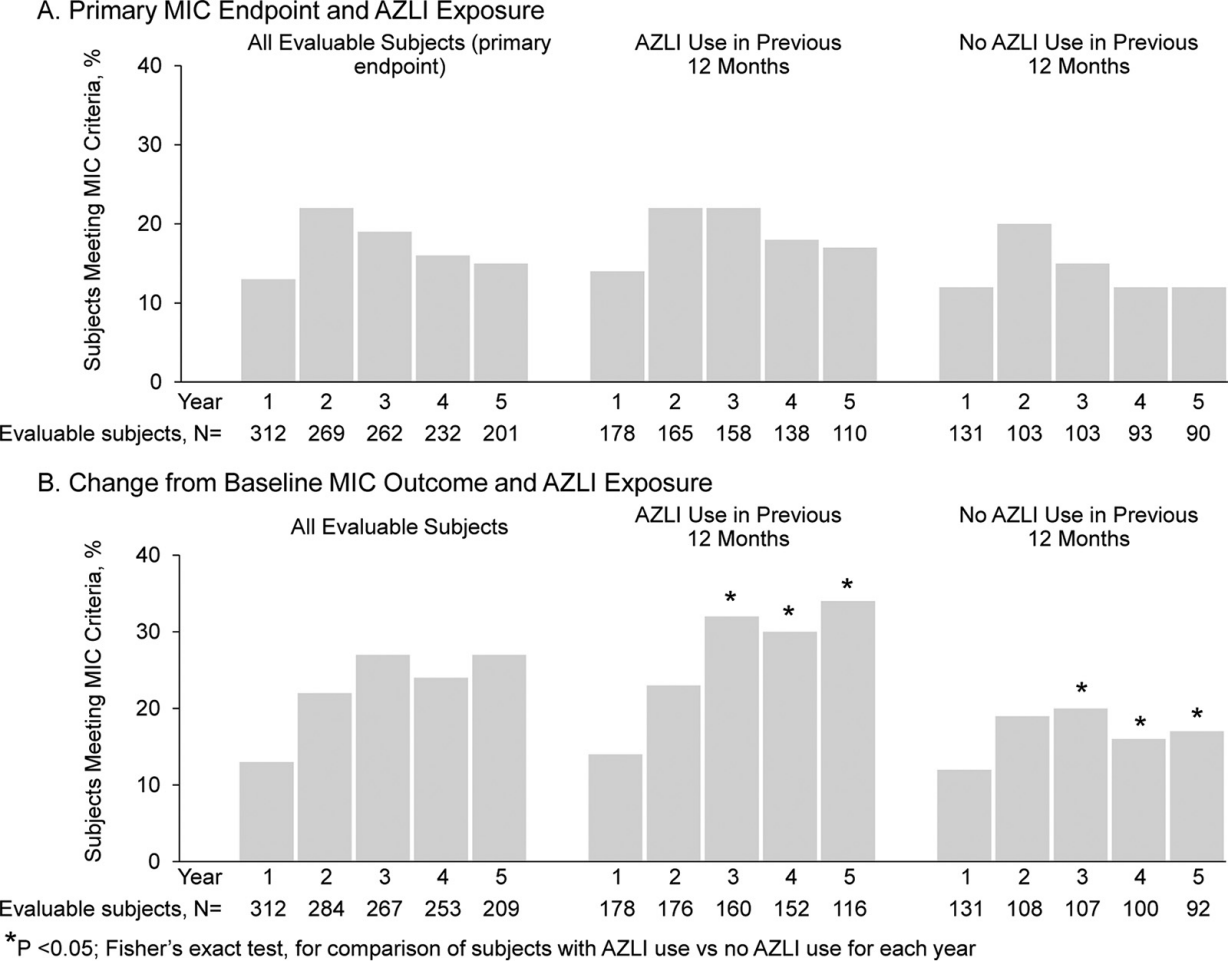
Changes in aztreonam susceptibility of Pseudomonas aeruginosa isolates. (A) Proportions of evaluable subjects who met the primary endpoint overall and by AZLI use in the previous 12 months (yes/no). The primary endpoint was met in a reporting year if the aztreonam MIC for the least susceptible *P. aeruginosa* isolate for that year was >8 μg/ml and had increased ≥4-fold compared with the MIC for the least susceptible isolate from the previous reporting year. (B) Proportions of evaluable subjects who met the secondary microbiologic outcome overall and by AZLI use in the previous 12 months (yes/no). The secondary microbiologic outcome was met in a reporting year if the aztreonam MIC for the least susceptible *P. aeruginosa* isolate for that year was >8 μg/ml and had increased ≥4-fold compared with the MIC for the least susceptible isolate observed at the baseline year. Data regarding AZLI use were not available for all subjects for either assessment; therefore, the numbers of evaluable subjects with and without AZLI use are usually slightly smaller than the total number of evaluable subjects. Note that subjects could move between AZLI use categories (yes/no for each year) across the study. *P* values were determined with Fisher’s exact test and are presented for descriptive purposes only.

The proportion of evaluable subjects whose least susceptible *P. aeruginosa* isolate met the primary endpoint remained consistent during the study (13 to 22% for 5 annual visits) (Fig. 1A) and was not associated with AZLI use during the previous 12 months. The primary endpoint was met by 14 to 22% of subjects with AZLI use versus 12 to 20% of subjects without AZLI use (*P* ≥ 0.05 for each year) (Fig. 1A).

### Secondary microbiologic outcome.

Comparing the MICs at the baseline year with those at each of the 5 annual study visits, 13% of evaluable subjects at year 1 and 22 to 27% at years 2 to 5 had a *P. aeruginosa* isolate with an MIC of >8 μg/ml that increased ≥4-fold at a reporting year visit (Fig. 1B). AZLI use was associated with a higher proportion of subjects meeting this secondary microbiologic outcome, compared with no AZLI use (*P* < 0.05 for years 3 to 5) (Fig. 1B).

### Exploratory microbiologic outcomes.

The proportion of evaluable subjects whose least susceptible *P. aeruginosa* isolate had an MIC above the aztreonam parenteral susceptibility breakpoint (>8 μg/ml) increased during the 5-year study, from 39% (*n* = 150/390) at enrollment to 37% (*n* = 132/353), 46% (*n* = 149/321), 49% (*n* = 151/308), 44% (*n* = 123/283), and 49% (*n* = 116/236) at years 1 to 5, respectively. However, aztreonam MIC_50_ and MIC_90_ values for all *P. aeruginosa* isolates changed ≤2-fold during the study (Table 3). At baseline, aztreonam MIC_50_ and MIC_90_ values were higher for isolates from subjects receiving AZLI during the previous 12 months than for subjects not receiving AZLI (8 and 256 μg/ml versus ≤1 and 32 μg/ml) (Table 3). This difference persisted during the 5-year study. For other antipseudomonal antibiotics tested, MIC_50_ and MIC_90_ values also increased ≤2-fold during the study (Table S1).

### Clinical outcomes by microbiologic endpoints.

**FIG 2:**
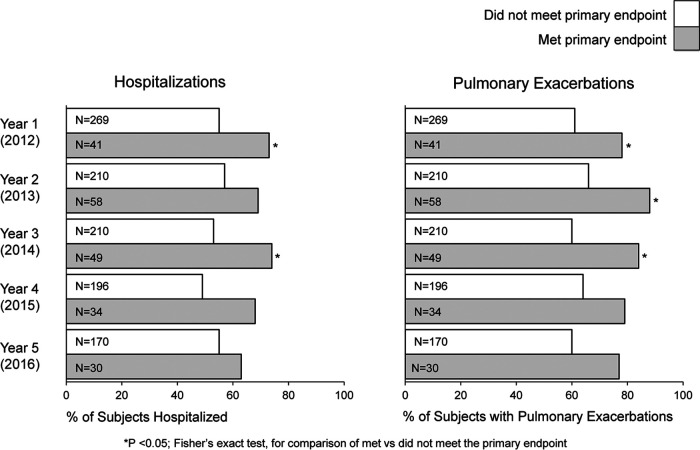
Hospitalizations and pulmonary exacerbations by primary microbiologic endpoint. The proportions of evaluable subjects who were hospitalized or had a pulmonary exacerbation each year were compared between groups who met or did not meet the primary endpoint for each year. Hospitalization and/or pulmonary exacerbation data were not available for every subject for every year; therefore, the total number of subjects evaluated in a given year (N) is smaller than the number of subjects evaluated for the primary endpoint, as shown in Table 2.

**FIG 3:**
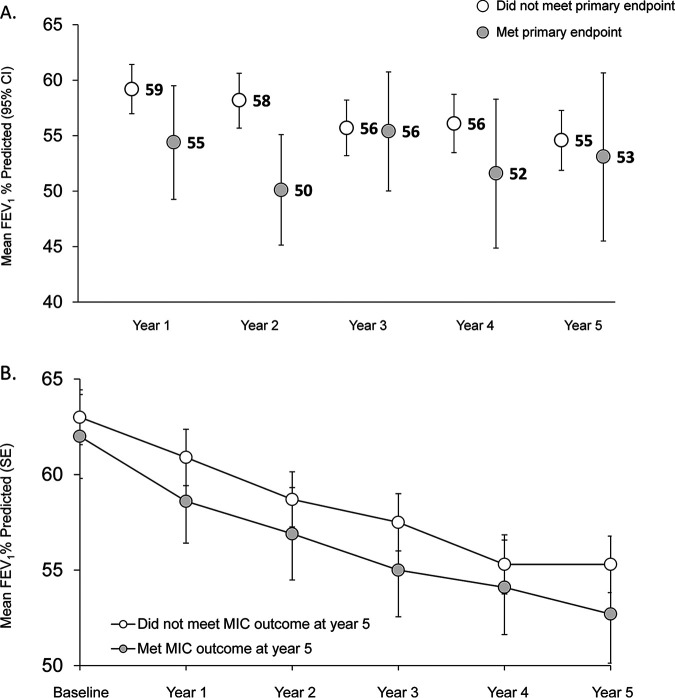
Changes in FEV_1_% predicted by primary microbiologic endpoint (A) and by secondary microbiologic outcome at year 5 (B). See Table 2 for a description of microbiologic outcomes. (A) Mean annualized FEV_1_% predicted by primary microbiologic endpoint at each reporting year. CI, confidence interval. (B) Mean annualized FEV_1_% predicted across the study for subjects who met or did not meet the secondary microbiologic outcome at reporting year 5. Note that subjects could move between AZLI use categories (yes/no for each year) across the study. SE, standard error.

Significantly more subjects meeting the primary endpoint were hospitalized in years 1 and 3 and had more pulmonary exacerbations in years 1, 2, and 3 than subjects not meeting the primary endpoint (Fig. 2). The annualized FEV_1_% predicted was lower for subjects meeting the primary microbiologic endpoint than for subjects not meeting it, although 95% confidence intervals (CIs) overlapped each year except for year 2 (Fig. 3A).

Pulmonary exacerbations were more common for subjects who met the secondary microbiologic outcome at year 5 (*n* = 54/56; 96%) than for subjects who did not meet it (*n* = 137/152; 90%); the median annual numbers of exacerbations (ranges) were 1.5 (0, 6.2) and 0.8 (0, 8.2), respectively (*P* < 0.05). Similarly, those who met the secondary outcome in year 5 had more hospitalizations (median, 1.2; range, 0, 6.8) than those who did not meet this outcome (median, 0.7; range 0, 8.4 [*P* < 0.05]). Mean FEV_1_% predicted values were consistently lower for those who met the secondary outcome at year 5 than for those who did not (Fig. 3B). The adjusted mean changes in FEV_1_% predicted from baseline to year 5 were comparable for both groups (mean change [standard error {SE}] of −9.3% [1.5%] versus −9.2% [0.9%], respectively [*P* ≥ 0.05]), which correspond to average annual declines of 1.9% and 1.8%, respectively.

### Clinical outcomes associated with baseline characteristics.

To provide further insight into these differences in clinical outcomes, we performed *post hoc* analyses of clinical events during the year of enrollment (2011) for subjects meeting/not meeting the secondary microbiologic outcome at year 5 (2016). In 2011, pulmonary exacerbations were reported for 77% of subjects (*n* = 43/56) who subsequently met the secondary outcome in year 5, compared with 56% of subjects (*n* = 85/152) who had exacerbations in 2011 but did not meet this outcome (*P* < 0.05). In 2011, the mean number of exacerbations per subject was also higher for subjects meeting than for those not meeting the secondary outcome in year 5 (1.6 versus 1.0 [*P* < 0.05]). In 2011, hospitalizations occurred for 61% (*n* = 34/56) of subjects meeting this outcome at year 5, compared with 51% (*n* = 77/152) of subjects not meeting it (*P* < 0.05). In contrast, annualized FEV_1_% predicted values (2011) were comparable, as the mean FEV_1_% predicted (standard deviation [SD]) was 62.0% (16.3%) for 55 subjects subsequently meeting this outcome, compared with 63.0% (17.6%) for 151 subjects not meeting it in year 5.

### Lung function associated with AZLI use.

The mean annualized FEV_1_% predicted in the year of enrollment was lower for subjects who had used AZLI in the 12 months before study participation than for subjects with no use (difference, −7.4) (Table 1). Comparable differences by AZLI use were observed across reporting visits (−5.9, −8.0, −5.6, −7.2, and −8.7 for reporting years 1 through 5, respectively). At year 5, mean annualized FEV_1_% predicted values (SD) were 52.8% (16.1%) for subjects who had used AZLI in the previous 12 months (*n* = 140) versus 61.5% (20.7%) for subjects who had not (*n* = 165).

Overall, 378 of the 510 subjects (74%) received AZLI at least once during the study, some of whom received AZLI every year (*n* = 156/510; 31%) (Table S2). The mean annualized FEV_1_% predicted in 2011 and 2016 as well as the mean annual rates of decline of FEV_1_% predicted and mean annual numbers of pulmonary exacerbations and hospitalizations (2012 to 2016) were comparable for these 2 groups of subjects with documented but variable AZLI exposure. However, for subjects who never received AZLI during the study, the mean annualized FEV_1_% predicted values were higher and the mean annual rate of decline was lower than those observed for the AZLI use groups, and mean numbers of annualized pulmonary exacerbations and hospitalizations were lower.

## DISCUSSION

Following the approval of AZLI (Cayston) in 2010, this 5-year observational study tracked *P. aeruginosa* susceptibilities and key clinical outcomes, as derived from the CFFPR, and tracked AZLI use, in a representative cohort of the U.S. CF population with chronic *P. aeruginosa* infection. The selection of the primary endpoint (proportion of subjects whose least susceptible *P. aeruginosa* isolate MIC was above the parenteral susceptibility breakpoint of >8 μg/ml and increased ≥4-fold compared with isolates from the previous year) represented a measurable change in susceptibility to aztreonam. During each year of this 5-year study, 13 to 22% of subjects met the primary microbiologic endpoint, but there was no sustained increase in the proportion of subjects meeting it. While AZLI use was not associated with the primary endpoint, those who used AZLI during the previous 12 months were more likely to meet the secondary microbiologic outcome. Over time, the proportion of subjects whose least susceptible isolate had an aztreonam MIC of >8 μg/ml increased, although the overall decline in susceptibility to aztreonam and other antipseudomonal antibiotics for all *P. aeruginosa* isolates, measured by changes in MIC_50_ and MIC_90_, remained ≤2-fold throughout the study.

We recognize that parenteral susceptibility breakpoints are currently the only available standard that defines “resistance” of bacterial pathogens to specific antibiotics. However, there is increasing debate about both the relevance of parenteral susceptibility breakpoints when selecting antibiotics for CF patients and the association of resistance with clinical outcomes (15, 16, 33). This *in vitro* benchmark does not consistently predict the clinical success or failure of antibiotic treatment in general (17), nor for the treatment of pulmonary exacerbations (6, 18, 19) or chronic *P. aeruginosa* suppression (20–22) in CF. Furthermore, the drug concentrations obtained within the airway following aerosol delivery greatly exceed parenteral MIC breakpoint values (20, 23–25), and no established susceptibility breakpoints exist for inhaled antibiotics. Nonetheless, our study endpoint criteria, designed in 2011, not only fulfilled the postmarketing commitment to the FDA but also provide insight into assessing the associations of susceptibility changes (or lack thereof) with clinical outcomes and the potential impact of treatment bias.

Emergence of *in vitro* resistance (using parenteral breakpoints) is likely inevitable when managing CF patients with chronic *P. aeruginosa* infection due to antibiotic selective pressure compounded by a large burden of bacteria growing in biofilms. This is particularly true for patients with more advanced disease who experience recurring cycles of pulmonary exacerbations and antibiotic treatments; CFFPR data show that patients with moderate or severe lung disease have higher rates of pulmonary exacerbations than those with mild lung disease (5, 13). Prior pulmonary exacerbations requiring intravenous (i.v.) antibiotics have been shown to be strongly associated with the time to the next exacerbation (26). In support of this premise, we identified a subset of subjects who had lower lung function at study entry who were more likely to subsequently meet the primary and secondary outcomes and who also experienced more pulmonary exacerbations and more hospitalizations (and associated systemic antibiotic exposure).

However, the rates of lung function decline throughout the study remained comparable between subjects meeting and those not meeting the secondary microbiologic outcome at year 5. This is consistent with results from a large observational study, the Epidemiologic Study of Cystic Fibrosis, in which the acquisition of multiantibiotic-resistant *P. aeruginosa* was not associated with a significant change in the rate of FEV_1_ decline despite greater inhaled and oral antibiotic use in these patients (23). The authors of that study concluded that highly resistant strains may be markers for more severe disease and more intense antibiotic treatments but do not, in themselves, accelerate lung function decline. Consistent with the association of antibiotic exposure with increased frequencies of exacerbations and hospitalizations, those who used AZLI during the year prior to enrollment had lower lung function. During the study, AZLI use was also associated with increased hospitalizations and exacerbations in some study years.

Limitations of this study include incomplete available data regarding total antibiotic exposure (including all oral/i.v./inhaled courses, doses, and adherence), the substantial number of subjects who were unevaluable due to study discontinuation or incomplete sputum sample collections, and missing data for hospitalizations. Data on comorbidities and complete concomitant medication use were not collected in this observational, noninterventional study. We used treatment with i.v. antibiotics as a surrogate for pulmonary exacerbations, which does not capture milder exacerbations treated with oral antibiotics. Furthermore, almost one-half (44%) of the subjects moved between AZLI use categories, receiving AZLI in some study years and not in others. This complicates any longitudinal comparisons of study outcomes. Finally, data derived from this observational study are subject to confounding by indication and therefore cannot be used to determine causality.

In conclusion, this study highlights the complexities of assessing relationships among inhaled aztreonam use, decreases in *P. aeruginosa in vitro* susceptibilities, and clinical outcomes. Decreased susceptibility to aztreonam, as measured by our microbiologic outcomes, was nominal over 5 years in our study population as a whole but appeared to be associated with greater antibiotic exposure and more frequent exacerbations and hospitalizations in some years. This investigation also successfully integrated CFFPR real-world clinical information with data derived during a 5-year prospective, observational study. Utilizing CFFPR data could further facilitate clinical research as novel therapies continue to emerge.

## MATERIALS AND METHODS

### Study design and subjects.

This observational, noninterventional 5-year study (August 2011 to December 2016) (ClinicalTrials.gov identifier NCT01375036) was conducted at 31 geographically distributed U.S. Cystic Fibrosis Foundation (CFF)-accredited care centers. Eligible subjects were participating in the CFFPR, were ≥6 years of age, had a FEV_1_% predicted of 25 to 90% at screening, and had ≥2 *P. aeruginosa*-positive respiratory tract cultures any time before enrollment. During the study, subjects received routine treatment provided by their CF care team. Subjects could participate in other clinical studies at the discretion of the investigators. The planned enrollment was 500 subjects, including at least 100 subjects with previous AZLI use, defined as postapproval prescriptions, in order to provide a representative cross-section of the CF population.

This study was conducted in accordance with recognized international scientific and ethical standards. Institutional review boards from each site approved the study. Patients/parents provided written informed consent/assent for study participation and use of their CFFPR data.

### Respiratory specimen collection and microbiologic processing.

Sputum samples or, if necessary, oropharyngeal swabs were collected at enrollment (2011) and each subsequent year (2012 to 2016). Consistent timing of collections between August and November was initially recommended, but the collection window was subsequently extended through December to maximize specimen collection. A central laboratory (Covance, Indianapolis, IN) processed respiratory specimens using American Society for Microbiology standards (27, 28). Specimens were plated on MacConkey agar and tryptic soy agar with 5% sheep blood and incubated for 48 h at 35°C. *P. aeruginosa* morphotypes (1 to 5/specimen) underwent susceptibility testing using microtiter plates containing serial 2-fold dilutions of antibiotics within the testing ranges specified by the Clinical and Laboratory Standards Institute for MIC analyses (29). Susceptibility testing was performed for aztreonam, cefepime, ceftazidime, ciprofloxacin, colistin, meropenem, piperacillin/tazobactam, and tobramycin.

### Cystic Fibrosis Foundation Patient Registry.

The CFFPR contains data from >30,000 U.S. pediatric and adult CF patients receiving care at CFF-accredited centers (1). The CFFPR provided annualized subject data (i.e., for each calendar year) for pulmonary exacerbations (defined as receiving i.v. antibiotics), the number of hospitalizations for any reason, and the mean FEV_1_% predicted. In 2012, Global Lung Function Initiative equations were adopted by the CFFPR to calculate FEV_1_% predicted (30).

### Study endpoints.

The primary study endpoint was the proportion of subjects whose least susceptible *P. aeruginosa* isolate had an aztreonam MIC that (i) was above the aztreonam parenteral breakpoint (>8 μg/ml) at a reporting year visit and (ii) increased ≥4-fold compared with the least susceptible *P. aeruginosa* isolate from the previous year visit. The latter criterion has been a commonly accepted standard for a significant change in the MIC value in previous CF studies (20, 31) and reflects more robust changes in the MIC than those demonstrated by 2-fold increases, which are often not considered to be clinically meaningful. Assessment of the primary endpoint required annual susceptibility data from 2 successive years (Table 2, upper series of gray bars). For example, the primary endpoint would be met for year 3 if the aztreonam MIC for a subject’s least susceptible *P. aeruginosa* isolate was 2 μg/ml in year 2 and 16 μg/ml in year 3.

The secondary microbiologic outcome was the proportion of subjects whose least susceptible *P. aeruginosa* isolate had an aztreonam MIC that (i) was above the parenteral breakpoint (>8 μg/ml) at the reporting year visit and (ii) increased ≥4-fold compared with the least susceptible *P. aeruginosa* isolate from the baseline visit (Table 2, lower series of gray bars). For example, the secondary outcome would be met for year 4 if the aztreonam MIC for a subject’s least susceptible isolate was 2 μg/ml at baseline and 16 μg/ml in year 4. The primary endpoints and secondary outcomes were compared for AZLI users (defined as receiving a ≥1-month-long course during the 12 months before a study visit as ascertained by a questionnaire at each study visit) versus nonusers.

Exploratory microbiologic outcomes included the annual proportion of subjects whose least susceptible *P. aeruginosa* isolate had an aztreonam MIC above the parenteral susceptibility breakpoint (>8 μg/ml) and potential changes in the annual MIC_50_ and MIC_90_ for aztreonam and other antipseudomonal antibiotics for all *P. aeruginosa* isolates. MIC_50_ and MIC_90_ were the MIC values at which 50% and 90% of isolates were inhibited, respectively. Changes in the MIC_50_ and MIC_90_ of aztreonam were compared for AZLI users versus nonusers.

Additionally, annualized CFFPR data were used to compare clinical outcomes between subjects meeting and those not meeting the primary or secondary microbiologic endpoint/outcome, including (i) the proportion of subjects experiencing pulmonary exacerbations, (ii) the proportion of subjects hospitalized, and (iii) lung function (mean FEV_1_% predicted and annual decline in FEV_1_% predicted). Mean annualized FEV_1_% predicted values were also compared for subjects with and those without AZLI use in the 12 months before a study visit.

Subjects could go on and off AZLI at any time during the study; AZLI use was determined by their CF care teams and not by the study protocol. Therefore, subjects could transition between the annual AZLI use categories (yes/no for each year) over the 5-year study.

### Statistical analyses.

A sample size of 500 subjects was selected in anticipation of having available data from ≥300 subjects for 5 years. Assuming a variability estimate of 0.25, a sample size of 300 would allow estimating the proportion of subjects who met the primary endpoint with a 95% CI of ±0.057. No formal hypotheses were tested; *P* values are presented for descriptive purposes only. No imputations were made for missing data. Differences in the percentages of subjects meeting and those not meeting study microbiologic endpoints were compared using Fisher’s exact test. Changes in FEV_1_% predicted were analyzed using analysis of covariance (ANCOVA) methods with baseline values for the reporting period and age at enrollment as covariates. The annual rate of decline in FEV_1_% predicted was estimated from a mixed model for repeated measures in a *post hoc* analysis. The numbers of hospitalizations and pulmonary exacerbations among those meeting and those not meeting the primary endpoint were compared by Poisson regression methods. Statistical analyses were performed using SAS software (v9.3; SAS Institute, Cary, NC).

## Supplementary Material

Supplemental file 1
