# Supplementary material for: Pseudomonas aeruginosa Susceptibility Patterns and Associated Clinical Outcomes in People with Cystic Fibrosis following Approval of Aztreonam Lysine for Inhalation
Source: Antimicrob Agents Chemother. 2021 Feb 17;65(3):e02327-20. doi: 10.1128/AAC.02327-20 (PMC8092527; doi:10.1128/AAC.02327-20)
Supplement: Supplemental file 1 [file AAC.02327-20-s0001.pdf]

Supplemental Table 1. MIC_{50} and MIC_{90} of Aztreonam and Other Antipseudomonal Antibiotics for All *Pseudomonas aeruginosa* Isolates

	Enrollment (2011) N = 611 *PA* isolates[a]		Year 5 (2016) N = 406 *PA* isolates[b]	
Antibiotic	MIC_{50} (µg/mL)	MIC_{90} (µg/mL)	MIC_{50} (µg/mL)	MIC_{90} (µg/mL)
Aztreonam	4	128	8	256
Cefepime	8	64	16	128
Ceftazidime	2	128	4	256
Ciprofloxacin	1	8	2	8
Meropenem	0.5	16	1	32
Piperacillin/ Tazobactam	8/ 4	256/ 4	8/ 4	512/ 4
Colistin	1	2	1	2
Tobramycin	2	32	2	32

a Median (range) number of isolates per subject = 1 (1-4).
b Median (range) number of isolates per subject = 2 (1-5).
MIC= minimum inhibitory concentration; MIC_{50} and MIC_{90} = MIC at which 50% and 90% of isolates are inhibited, respectively; *PA* = *Pseudomonas aeruginosa*

Supplemental Table 2. FEV_1 % predicted, pulmonary exacerbations, and hospitalizations for subjects in different AZLI-use categories.

	Exposed to AZLI Every Year[a] (N = 156)	Exposed to AZLI at Least Once[a] (N = 378)	Never Exposed to AZLI[a] (N = 132)
Annualized FEV_1 % predicted for 2011,[b] mean (SD)	59.8 (17.4)	61.8 (18.8)	69.0 (17.4)
Annualized FEV_1 % predicted for 2016,[c] mean (SD)	53.4 (15.6)	54.5 (18.4)	65.3 (20.6)
Annual rate of decline of FEV_1 % predicted, 2011-2016,[b, d] LS mean (SE)	-2.0 (0.1)	-2.2 (0.1)	-1.4 (0.1)
Annual number of pulmonary exacerbations, 2012-2016,[e] mean (SD)	1.6 (1.3)	1.6 (1.4)	1.1 (1.3)
Annual number of hospitalizations, 2012-2016,[e] mean (SD)	1.3 (1.3)	1.4 (1.4)	1.1 (1.3)
Annual number of hospitalization days, 2012-2016,[e] mean (SD)	18.1 (23.0)	17.2 (22.5)	14.7 (29.1)

a AZLI use was defined as receiving > 1-month-long course during the 12 months before a study visit as ascertained by a questionnaire at each study visit. A total of 222 subjects moved between AZLI-use categories (yes/no each year) across the study.

b CFF Registry data available for 155, 375, and 128 subjects for the 3 columns, respectively.

c CFF Registry data available for 93, 251, and 84 subjects for the 3 columns, respectively.

d Annual rate of decline in FEV_1 % predicted estimated from mixed model for repeated measures in a post hoc analysis.

e CFF Registry data available for 155, 375, and 126 subjects for the 3 columns, respectively.

CFF = Cystic Fibrosis Foundation; FEV_1 = forced expiratory volume in 1 second; LS = least-squares; SD = standard deviation; SE = standard error

Supplemental Figure 1.

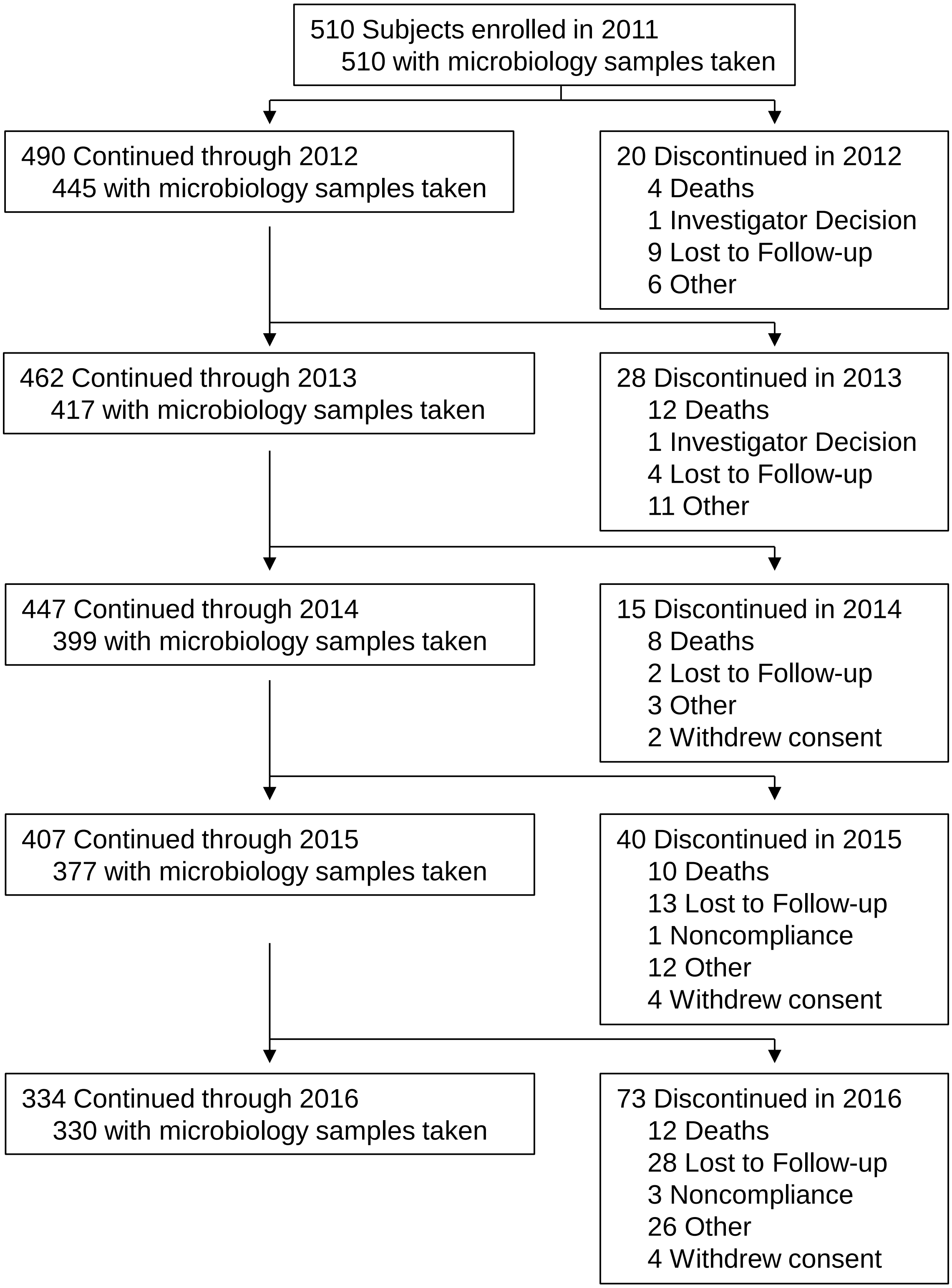

9
